# Advanced lung cancer inflammation index and its prognostic value in HPV-negative head and neck squamous cell carcinoma: a multicentre study

**DOI:** 10.1007/s00520-020-05979-9

**Published:** 2021-01-29

**Authors:** Piergiorgio Gaudioso, Daniele Borsetto, Giancarlo Tirelli, Margherita Tofanelli, Fiordaliso Cragnolini, Anna Menegaldo, Cristoforo Fabbris, Gabriele Molteni, Daniele Marchioni, Piero Nicolai, Paolo Bossi, Andrea Ciorba, Stefano Pelucchi, Chiara Bianchini, Simone Mauramati, Marco Benazzo, Vittorio Giacomarra, Roberto Di Carlo, Mantegh Sethi, Jerry Polesel, Jonathan Fussey, Paolo Boscolo-Rizzo

**Affiliations:** 1 Department of Neurosciences, Section of Otolaryngology, University of Padova, Treviso, Italy; 2 Department of ENT, Addenbrooke’s Hospital, Cambridge University Hospitals NHS Foundation Trust, Cambridge, UK; 3 Department of Medical, Surgical and Health Sciences, Section of Otolaryngology, University of Trieste, Trieste, Italy; 4 Section of Ear Nose and Throat (ENT), Department of Surgical Sciences, Dentistry, Gynecology and Pediatrics, University of Verona, Verona, Italy; 5 Department of Otorhinolaryngology—Head Neck Surgery, University of Brescia, Brescia, Italy; 6 Medical Oncology Unit, Department of Medical Oncology, ASST Spedali Civili di Brescia, Brescia, Italy; 7 ENT Department, University Hospital of Ferrara, Ferrara, Italy; 8 Department of Otorhinolaryngology, University of Pavia, IRCCS Policlinico “San Matteo” Foundation, Pavia, Italy; 9 Unit of Otolaryngology, Azienda Ospedaliera “S. Maria degli Angeli”, Pordenone, Italy; 10 Department of Neurosciences, Section of Otolaryngology, University of Padova, Padova, Italy; 11 Department of Otolaryngology, Leeds University Hospital, Leeds, UK; 12 Unit of Cancer Epidemiology, Centro di Riferimento Oncologico di Aviano (CRO) IRCCS, Aviano, Italy; 13 Department of ENT/Head and Neck Surgery, Queen Elizabeth University Hospital Birmingham, Birmingham, UK

**Keywords:** Head and neck cancer, Nutrition, Advanced lung cancer inflammation index, Inflammatory indexes, Survival

## Abstract

**Purpose:**

The aim of this study is to evaluate the prognostic value of pre-treatment advanced lung cancer inflammation index (ALI) in patients with HPV-negative HNSCC undergoing up-front surgical treatment.

**Methods:**

The present multi-centre, retrospective study was performed in a consecutive cohort of patients who underwent upfront surgery with or without adjuvant (chemo)-radiotherapy for head and neck squamous cell carcinoma (HNSCC). Patients were stratified by ALI, and survival outcomes were compared between groups. In addition, the prognostic value of ALI was compared with two other indices, the prognostic nutritional index (PNI) and systemic inflammatory index (SIM).

**Results:**

Two hundred twenty-three patients met the inclusion criteria (151 male and 72 female). Overall and progression-free survival were significantly predicted by ALI < 20.4 (HR 3.23, CI 1.51–6.90 for PFS and HR 3.41, CI 1.47–7.91 for OS). Similarly, PNI < 40.5 (HR = 2.43, 95% CI: 1.31–4.51 for PFS and HR = 2.40, 95% CI: 1.19–4.82 for OS) and SIM > 2.5 (HR = 2.51, 95% CI: 1.23–5.10 for PFS and HR = 2.60, 95% CI: 1.19–5.67 for OS) were found to be significant predictors. Among the three indices, ALI < 20.4 identified the patients with the worst 5-year outcomes. Moreover, patients with a combination of low PNI and low ALI resulted to be a better predictor of progression (HR = 5.26, 95% CI: 2.01–13.73) and death (HR = 5.68, 95% CI: 1.92–16.79) than low ALI and low PNI considered alone.

**Conclusions:**

Our results support the use of pre-treatment ALI, an easily measurable inflammatory/nutritional index, in daily clinical practice to improve prognostic stratification in surgically treated HPV-negative HNSCC.

## Introduction

In recent years, the role of the inflammatory system and immunity in head and neck squamous cell carcinoma (HNSCC) tumorigenesis has been the subject of intense interest among researchers, with the focus being primarily on tumor infiltrating immune cells (TIICs) and immunoediting mechanisms [1, 2].

Several subtypes of TIICs have been observed to be associated with HNSCC prognosis and treatment response [3]. Interestingly, tumors seem to induce systemic immune changes in peripherical blood cells in order to promote cancer progression [4], meaning that immune cells in the peripheral blood could be as important as those in the tumor microenvironment (TME) [5]. This is consistent with a systemic disease interpretation of cancer. The tumor-associated immune landscape may be reflected in peripheral blood leucocyte counts via the interaction between tumor cells, TIICs, and stromal cells, which promotes proinflammatory cytokine production (i.e., TNFα, interleukins, TGFβ, CXCLs) leading to immune cell recruitment, as has been described in the case of neutrophils and monocytes [6, 7]. Evidence for specific mechanisms are now emerging, for example the description of raised granulocyte-colony stimulating factor (G-CSF) in tumor development and consequent bone marrow reprogramming, with activation of a myeloid differentiation program in the early hematopoietic compartment, and an expansion of T cell-suppressive myeloid cells [8]. These mechanisms are yet to be fully understood, although their possible clinical implications are significant, as evidenced by the finding that lower lymphocyte and higher platelet, neutrophil, and monocyte counts are associated with poor prognosis [9].

Several inflammatory indices based on peripherical white blood cell counts, including lymphocyte-to-neutrophil ratio (LNR) [10], systemic inflammatory marker (SIM) [11], prognostic nutritional index (PNI) [12], and H-Index [13], have been proposed to provide health researchers with more comprehensive and accurate prognostication.

In recent years, inflammatory indices have been supplemented with nutritional information to produce novel indices. Among them, the advanced lung cancer inflammation index (ALI) is a novel prognostic index designed for metastatic non-small cell lung cancer (NSCLC) and also found to be associated with OS in small cell lung cancer, large B cell lymphoma, esophageal squamous carcinoma, and colorectal cancer [14]. Described for the first time in 2013 by Jafri et al. [15], ALI is calculated using neutrocyte-to-lymphocyte ratio (NLR) as well as body mass index (BMI) and baseline serum albumin. Thus, ALI includes both inflammatory and nutritional aspects, the latter being another well-investigated prognostic factor for HNSCC [16]. The prognostic value of ALI in HNSCC has been observed only in one study and found to predict both overall and disease-free survival [17]; however, the cohort was small, and no comparison was made between ALI and other inflammatory indices.

The aim of this study is to evaluate the prognostic value of pre-treatment ALI in patients with HPV-negative HNSCC undergoing up-front surgical treatment, and to compare its prediction accuracy with two other indices, the PNI and SIM.

## Methods

The present multi-centre, retrospective study was performed in a cohort of consecutive patients diagnosed with HNSCC who underwent up-front surgery and met the inclusion criteria from April 2004 to April 2017. The study network included General and University Hospitals in north-eastern Italy, located in Treviso, Padova, Verona, Trieste, Brescia, Pordenone, Ferrara, and Pavia. Inclusion criteria were (a) HNSCC arising from the oral cavity, oropharynx, hypopharynx, or larynx; (b) curative up-front surgery as the primary treatment modality; and (c) availability of body mass index (BMI) and blood parameters for ALI calculation. Patients were specifically excluded if (a) they were diagnosed with nasopharyngeal carcinoma or T1 glottic SCC; (b) they had any coexisting conditions or hematological conditions that could alter inflammatory parameters; (c) they had previous malignancy or additional synchronous primary tumors; (d) their pre-treatment blood test results were not available; (e) they had metastatic disease; and (f) HPV-driven SCC.

### Participants and data

Medical records were reviewed to collect socio-demographic and clinical characteristics of enrolled patients. Baseline characteristics, including body mass index (BMI), Adult Comorbidity Evaluation 27 (ACE-27) comorbidity index, clinical stage, histology, and grading were retrieved. For oropharyngeal carcinomas, HPV status was assessed by p16 immunostaining and/or HPV-DNA. Blood parameters were collected at baseline and before treatment, including red blood cell (10^3^/μL), white blood cell (10^3^/μL), platelet (10^3^/μL), hemoglobin (Hb, g/L), hematocrit (%), mean corpuscular volume (MCV, fL), mean platelet volume (MPV, fL), neutrophils (10^3^/μL), lymphocytes (10^3^/μL), monocytes (10^3^/μL), basophils (10^3^/μL), eosinophils (10^3^/μL), serum albumin (g/dL), and C-reactive protein (CRP).

Patients were routinely followed-up according to consensus guidelines [18] with endoscopic examination of the upper aerodigestive tract every 1–3 months for the first year, 3–4 months during the second year, 4–6 months during the 3rd year, and every 6 months after that. A dedicated CT scan of the chest was done annually. Additional dedicated head and neck imaging was arranged based on clinical features and local protocol.

### Inflammatory indices

Pre-treatment ALI [15], PNI [12], and SIM [11] indices were calculated as illustrated in Appendix Table 5.

### Statistics

Median values of blood markers and corresponding interquartile ranges (Q1–Q3) were reported; differences in blood markers across socio-demographic and clinical characteristics were evaluated through the Kruskal-Wallis test.

For each patient, the time at risk was computed from the date of surgery to the date of locoregional recurrence, death, or last follow-up, whichever occurred first according to the outcome of interest. xProgression-free survival (PFS) was defined as the time from surgery to any type of recurrence/progression or death from any cause. Overall survival (OS) was defined as the time from surgery to death from any cause. The Kaplan-Meier method was used to generate crude survival probabilities and the log-rank test was used to assess the heterogeneity in time to event in strata of selected covariates [19], censoring follow-up at 10 years. Hazard ratios (HR) and the corresponding 95% CI were calculated using Cox proportional hazards models [19], adjusting for gender and age, plus covariates significantly associated to OS in the multivariable analysis (i.e., education and cN). ALI, PNI, and SIM were categorized in three levels; the optimal cut-offs were determined according to a recursive algorithm that maximizes the model predictability in OS, measured through Harrell’s C-index [20].

## Results

### Population

Overall, 223 patients met the inclusion criteria (median age, 66 years; interquartile range, 59–73 years); the majority of patients (*n* = 151, 67.7%) were male, with stage III–IV cancer (*n* = 158, 63.7%) and with moderately differentiated SSC (*n* = 155, 69.5%; Table 1). Negative surgical margins were achieved in 176 patients (78.9%) and extracapsular extension was absent in 177 patients (89.4%). Adjuvant (chemo)radiotherapy was administered to 98 patients (43.9%).

**Table 1:** Hazard ratio (HR) and corresponding confidence interval (CI) for loco-regional failure, progression, and death according to socio-demographic and clinical characteristics

	Patients		Locoregional failure	PFS	OS
	*n*	(%)	HR (95% CI)^a^	HR (95% CI)	HR (95% CI)
Gender					
Male	151	(67.7)	Reference	Reference	Reference
Female	72	(32.3)	0.81 (0.32–2.05)	0.77 (0.44–1.36)	0.68 (0.36–1.29)
Age (years)					
< 65	89	(39.9)	Reference	Reference	Reference
65–74	52	(23.3)	0.72 (0.26–2.04)	1.51 (0.78–2.92)	2.09 (0.95–4.58)
≥ 75	82	(36.8)	0.41 (0.15–1.15)	1.24 (0.65–2.34)	2.10 (1.00–4.42)
Education^b^					
Low	151	(70.9)	Reference	Reference	Reference
High	62	(29.1)	0.45 (0.17–1.20)	0.46 (0.23–0.92)	0.38 (0.16–0.90)
BMI (kg m^−2^)					
< 25	124	(55.6)	Reference	Reference	Reference
≥ 25	99	(44.4)	1.34 (0.56–3.19)	0.93 (0.55–1.57)	0.81 (0.44–1.48)
Smoking habits^c^					
Never	42	(19.3)	Reference	Reference	Reference
Ever	176	(80.7)	1.36 (0.38–4.82)	1.12 (0.55–2.25)	0.96 (0.45–2.02)
Drinking habits^d^					
Never	122	(61.3)	Reference	Reference	Reference
Ever	77	(38.7)	0.53 (0.21–1.33)	0.73 (0.42–1.28)	0.69 (0.36–1.30)
ACE-27					
None-Mild	101	(45.3)	Reference	Reference	Reference
Moderate-Severe	122	(54.7)	0.83 (0.30–2.35)	1.17 (0.66–2.05)	1.00 (0.53–1.89)
Cancer site					
Oral cavity	109	(47.4)	Reference	Reference	Reference
Oropharynx	26	(11.3)	7.93 (2.47–25.50)	2.01 (0.99–4.07)	1.11 (0.47–2.60)
Hypopharynx/Larynx	95	(41.3)	0.53 (0.14–2.08)	1.01 (0.55–1.86)	1.14 (0.59–2.21)
pT					
pT1-pT2	104	(46.6)	Reference	Reference	Reference
pT3-pT4	119	(53.4)	0.51 (0.22–1.14)	0.81 (0.46–1.41)	1.11 (0.59–2.09)
pN^e^					
pN0	143	(65.0)	Reference	Reference	Reference
pN1-pN3	77	(35.0)	1.34 (0.56–3.24)	2.00 (0.16–3.44)	2.35 (1.28–4.32)
Stage					
I–II	90	(36.3)	Reference	Reference	Reference
III–V	158	(63.7)	0.54 (0.22–1.31)	1.02 (0.58–1.79)	1.35 (0.70–2.61)
Grading (differentiation)					
Well	22	(9.9)	Reference	Reference	Reference
Moderately	155	(69.5)	1.32 (0.17–10.46)	1.35 (0.41–4.49)	1.52 (0.35–6.54)
Poorly	46	(20.6)	1.72 (0.20–15.10)	2.34 (0.65–8.45)	2.30 (0.49–10.85)
Surgical margins					
Negative	176	(78.9)	Reference	Reference	Reference
Close/Positive	47	(21.1)	2.45 (1.00–5.97)	1.76 (0.99–3.12)	1.96 (1.04–3.69)
Extracapsular extension					
Absent	177	(89.4)	Reference	Reference	Reference
Present	21	(10.6)	1.27 (0.57–2.83)	1.36 (0.63–2.93)	1.14 (0.49–2.67)

HRs and CIs were estimated from Cox proportional hazard model, adjusting for gender, age, education, pN, and surgical margins

^a^Adjusted for competing risks according to Fine-Gray model

^b^Education level is missing in 10 patients

^c^Smoking habit is missing in five patients

^d^Drinking habit is missing in 24 patients

^e^pN is missing in three patients

During a median follow-up of 58 months (interquartile range, 41–83 months), 59 patients died; cancer was the cause of death for 31 (52.5%) of them. Local recurrence was experienced by 23 patients, while 21 patients had regional recurrence and 11 distant metastases. Second primary tumor was diagnosed during follow-up in 28 patients.

Among socio-demographic and clinical characteristics, a significant association emerged between higher education and improved both PFS (HR = 0.46, 95% CI: 0.23–0.92) and OS (HR = 0.38, 95% CI: 0.16–0.90; Table 1). Moreover, oropharyngeal primary site was associated with an increased risk of locoregional failure (HR = 7.93, 95% CI: 2.47–25.50), but not of progression or death. Positive surgical margins were associated with higher risk of locoregional failure (HR = 2.45, 95% CI: 1.00–5.97) and lower OS (HR for death 1.96, 95% CI: 1.04–3.69).

Blood samples were obtained a median (IQR) of 18 (10 to 27) days before surgery. Table 2 shows the correlations between blood parameters and cancer outcomes. Patients with serum albumin levels ≥ 4.4 g/dL reported a significant reduced risk of a PFS event (HR = 0.49, 95% CI: 0.25–0.94). Neutrophil, lymphocyte and monocyte counts were not predictors of locoregional failure, PFS, and OS.

**Table 2:** Hazard ratio (HR) and corresponding 95% confidence interval (CI) for local failure, regional failure, distant failure, progression, and death according to blood parameters

	Pts	Loco-regional failure	PFS	OS
		HR (95% CI)^a^	HR (95% CI)	HR (95% CI)
Hemoglobin (g/L)				
< 139	75	Reference	Reference	Reference
139–151	89	1.50 (0.55–4.08)	0.75 (0.42–1.32)	0.56 (0.29–1.08)
≥ 152	59	0.80 (0.23–2.75)	0.43 (0.20–0.95)	0.41 (0.17–0.98)
Albumin (g/dL)				
< 4.0	75	Reference	Reference	Reference
4.0–4.3	74	0.80 (0.28–2.23)	0.59 (0.31–1.10)	0.59 (0.29–1.22)
≥ 4.4	74	0.88 (0.34–2.31)	0.49 (0.25–0.94)	0.53 (0.25–1.09)
Neutrophils (10^3^/μL)				
< 4.1	74	Reference	Reference	Reference
4.1–5.7	75	1.29 (0.38–4.32)	1.30 (0.65–2.57)	1.10 (0.52–2.34)
≥ 5.8	74	0.98 (0.31–3.07)	1.16 (0.58–2.33)	1.05 (0.48–2.28)
Lymphocytes (10^3^/μL)				
< 1.6	72	Reference	Reference	Reference
1.6–2.0	84	0.70 (0.24–2.06)	0.73 (0.39–1.35)	0.83 (0.42–1.63)
≥ 2.1	67	1.25 (0.45–3.44)	0.85 (0.43–1.67)	1.80 (0.36–1.74)
Monocytes (10^3^/μL)				
< 0.51	84	Reference	Reference	Reference
0.51–0.71	71	0.64 (0.19–2.20)	0.93 (0.47–1.82)	0.96 (0.46–1.98)
≥ 0.72	68	1.13 (0.42–3.05)	1.72 (0.91–3.26)	1.66 (0.78–3.50)

HRs and CIs were estimated from Cox proportional hazard model, adjusting for gender, age, education, pN, and surgical margins

^a^Adjusted for competing risks according to Fine-Gray model

### Inflammatory indices

Patients were stratified into 3 prognostic groups for each inflammatory index, with 3 ranges of values associated with higher, intermediate or lower survival. The optimal critical values to define these ranges were found to be 20.4 and 50.3 for ALI, 40.5 and 42.4 for PNI, and 1.3 and 2.5 for SIM.

**Fig. 1:**
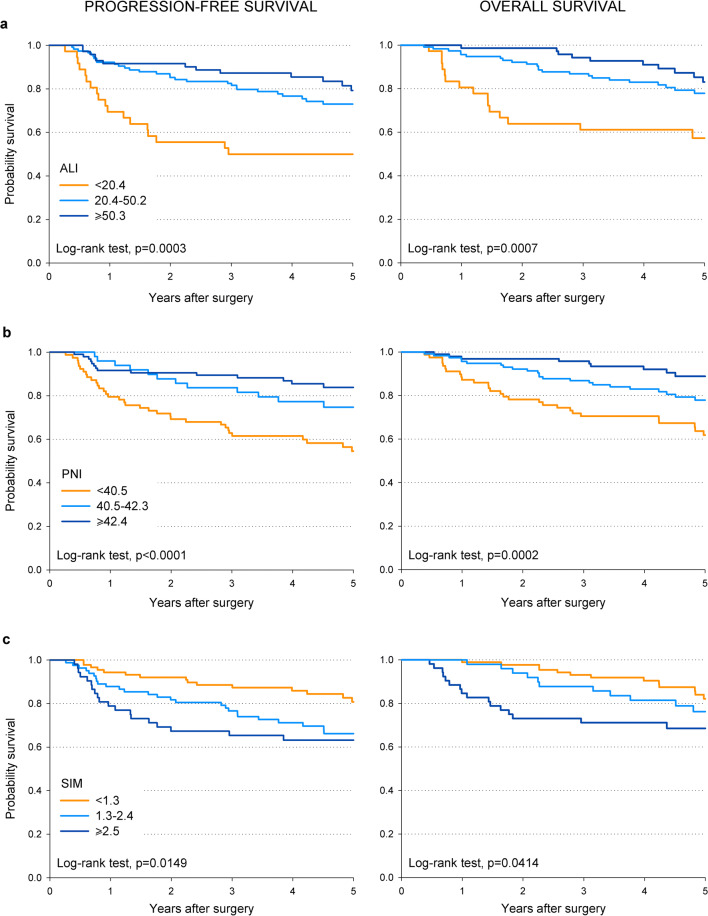
Kaplan-Meier curves showing the correlation of ALI, PNI, and SIM with the 5-year progression-free and overall survival

Patients with ALI < 20.4 reported the worst prognosis, with 5-year PFS of 50.0% compared to 79.3% in those with ALI ≥ 50.3 (*p* = 0.0003; Fig. 1). Similarly, 5-year OS was 57.3% and 83.1%, respectively (*p* = 0.0007; Fig. 1). The disadvantage in survival for patients with ALI < 20.4 was confirmed by multivariate analysis, with hazard ratios (HR) of 3.23 (95% CI: 1.51–6.90) for PFS and 3.41 (95% CI: 1.47–7.91) for OS (Table 3). Similar patterns emerged for PNI < 40.5 (HR = 2.43, 95% CI: 1.31–4.51 for PFS and HR = 2.40, 95% CI: 1.19–4.82 for OS) and SIM > 2.5 (HR = 2.51, 95% CI: 1.23–5.10 for PFS and HR = 2.60, 95% CI: 1.19–5.67 for OS). No index was significantly associated to loco-regional failure (Table 3).

**Table 3:** Hazard ratio (HR) and corresponding 95% confidence interval (CI) for loco-regional failure, progression, and death according to inflammatory and nutritional indexes

	Pts	Locoregional failure	PFS	OS
		HR (95% CI)^a^	HR (95% CI)	HR (95% CI)
NLR				
≥ 3.7	49	Reference	Reference	Reference
< 3.7	174	0.77 (0.30–1.96)	0.53 (0.30–0.92)	0.51 (0.28–0.96)
ALI				
≥ 50.3	71	Reference	Reference	Reference
20.4–50.2	116	0.83 (0.30–2.32)	1.32 (0.66–2.66)	1.30 (0.58–2.91)
< 20.4	36	1.31 (0.40–4.27)	3.23 (1.51–6.90)	3.41 (1.47–7.91)
PNI				
≥ 42.4	95	Reference	Reference	Reference
40.5–42.3	49	1.49 (0.48–4.62)	1.53 (0.74–3.16)	1.48 (0.64–3.41)
< 40.5	79	1.64 (0.65–4.11)	2.43 (1.31–4.51)	2.40 (1.19–4.82)
SIM				
< 1.3	88	Reference	Reference	Reference
1.3–2.4	83	1.39 (0.44–4.44)	1.67 (0.86–3.27)	1.35 (0.65–2.83)
≥ 2.5	52	2.24 (0.73–6.90)	2.51 (1.23–5.10)	2.60 (1.19–5.67)

HRs and CIs were estimated from Cox proportional hazard model, adjusting for gender, age, education, pN, and surgical margins

^a^Adjusted for competing risks according to Fine-Gray model

Interestingly, among the three indices, ALI < 20.4 identified the patients with the worst 5-year outcomes (i.e., 50.0% for PFS and 57.3% for OS; Fig. 1), whereas PNI ≥ 42.4 identified those with the best prognosis (i.e., 83.8% for PFS and 88.8% for OS; Fig. 1).

We therefore analyzed the combination of PNI and ALI in relation to prognosis (Table 4). Patients with low ALI and low PNI reported a risk of progression (HR = 5.26, 95% CI: 2.01–13.73) and death (HR = 5.68, 95% CI: 1.92–16.79) much higher than ALI and PNI considered alone. Interestingly, BMI, hemoglobin, albumin, and lymphocytes were significantly lower in patients with ALI < 20.4 and PNI < 40.5 compared to patients with ALI ≥ 42.4 and PNI ≥ 50.3 (Table 4). Conversely, neutrophils were significantly higher.

**Table 4:** Median values of baseline parameters and hazard ratio (HR) and corresponding confidence intervals (CI) of oncological outcomes in 223 patients with head and neck cancer undergoing surgery, according to combination of prognostic nutritional index (PNI) and advanced lung inflammation index (ALI)

	ALI ≥ 42.4 and PNI ≥ 50.3	20.4 ≤ ALI < 42.4 or 40.5 ≤ PNI < 50.4	ALI < 20.4 and PNI < 40.5	
	(*n* = 42)	(*n* = 156)	(*n* = 25)	
Oncological outcomes				
Loco-regional failure (HR, 95% CI)^a^	Reference	1.20 (0.30–4.74)	1.25 (0.25–6.35)	
Progression-free survival (HR, 95% CI)	Reference	1.43 (0.61–3.32)	5.26 (2.01–13.73)	
Overall survival (HR, 95% CI)	Reference	1.39 (0.52–3.75)	5.68 (1.92–16.79)	
Baseline parameters				
BMI (kg m^−2^)	26.6 (23.1–28.8)	24.1 (21.7–26.5)	23.8 (19.9–27.1)	*p* = 0.0014
Hemoglobin (g/L)	152 (140–160)	140 (130–150)	121 (117–140)	*p* < 0.0001
Albumin (g/dL)	4.50 (4.35–4.70)	4.10 (3.92–4.38)	3.40 (3.26–3.70)	*p* < 0.0001
Neutrophils (10^3^/μL)	3.95 (3.32–4.80)	4.68 (3.77–6.50)	6.90 (5.30–8.65)	*p* < 0.0001
Lymphocytes (10^3^/μL)	2.12 (1.84–2.35)	1.89 (1.40–2.22)	1.25 (0.90–1.46)	*p* < 0.0001
Monocytes (10^3^/μL)	0.60 (0.40–0.70)	0.60 (0.50–0.80)	0.68 (0.50–1.11)	*p* = 0.1482

HRs and CIs were estimated from Cox proportional hazard model, adjusting for gender, age, education, pN, and surgical margins

^a^Adjusted for competing risks according to Fine-Gray model

## Discussion

In the present study, we report the prognostic value of ALI in patients with HPV-negative HNSCC treated by upfront surgery. Irrespective of other stage-related prognostic parameters, a low ALI was associated with a poor prognosis.

To date, the most robust prognostic factor in head and neck oncology is HPV-status which was recently incorporated in the 8th edition of TNM staging system by its surrogate biomarker p16 [21]. However, its role is limited to oropharyngeal SCC, and reliable biomarkers for the stratification of prognosis in non-oropharyngeal HNSCC and HPV-negative oropharyngeal SCC are lacking. For these reasons and in order to study a more homogeneous population, we selected for the present analysis only HPV-negative HNSCCs.

Inflammatory indices are a prognostic tool that reflects the immunity of the host response to cancer progression. Cancer influences the immune system in a pro-tumorigenic way, increasing neutrophil and monocyte count and decreasing lymphocyte count. The former are involved in tumor initiation, growth, proliferation, or metastasis, the latter suppresses tumor development and growth through immune surveillance mechanisms [11]. An association between higher ALI and survival has been described in several different cancer types (non-small cell lung cancer, small cell lung cancer, diffuse large B cell lymphoma, and colorectal cancer) [14] but until now has only been reported in HNSCC by a small-cohort retrospective study, which found a correlation with prognosis [11].

Among standard socio-demographic and clinical parameters, only age, low educational status, neck node metastases, and close/positive margins were independently associated with worse OS in the present study. However, in addition, we identified a significant independent association between several inflammatory/nutritional indices, including lower ALI, PNI, and higher SIM with both inferior PFS and OS. Of these, the ALI was found to be a more reliable prognostic index, with stronger associations with PFS and OS compared with PNI and SIM. This may be due to the more complete representation and synthesis of the inflammatory and nutritional status of the patient. We also found that higher serum albumin level was the only blood parameter significantly associated with a better PFS when considered alone, which highlights the importance of preoperative nutritional status and the potential value of nutrient supplementation [16]. Particularly, early nutrition intervention in patients with HNSCC was observed to result in an improved treatment tolerance and outcome [22].

Moreover, patients with both an ALI < 20.4 and a PNI < 40.5 had a significantly lower PFS and OS than those with a low score on either index alone. The reason for this synergy between ALI and PNI is unclear, given the common parameters composing ALI and PNI.

ALI has been previously investigated in HNSCC in only one small single-centre retrospective study [17]. The present multicentre study provides a relatively large cohort of highly selected patients with strict inclusion criteria. Follow-up was accurate, with regular clinical radiological examination as recommended by the American Cancer Society [23]. Despite these strengths, this study does also have some limitations. Firstly, the retrospective design may have biased the results. Secondly, blood parameters were collected pre-operatively in order to avoid the influence of surgery itself on the baseline values. However, it was not always possible to exclude the influence of any other systemic condition (such as inflammatory or infectious conditions) as ALI, PNI, and SIM are nonspecific tumor markers. Thirdly, 122 patients reported abstinence from alcohol, which does not reflect the drinking prevalence of the region. Finally, given the period of time during which included patients were diagnosed and treated, HNSCC were staged according to the 7th edition of the AJCC TNM. However, considering that we excluded HPV-positive patients, the discrepancy between the 7th and the 8th editions of AJCC TNM was limited.

The present study supports the use of ALI as a prognostic marker, offering evidence of a strong correlation with prognosis. ALI can be easily calculated in routine clinical practice using standard blood tests and clinical parameters to help inform clinicians and patients on prognosis. Further research is required to confirm the usefulness of blood parameters in the assessment of inflammatory and nutritional status, and their importance in prognostication and eventually perhaps in therapeutic strategy.

At present, the AJCC TNM staging system for HNSCC includes only tumor-associated factors in risk stratification. However, heterogeneity is still evident within staging groups, as reflected by the present analysis. The encouraging performance of inflammatory and nutritional indices in stratifying outcomes in patients with HNSCC supports further large and prospective research to verify whether the integration of host-related factors with tumor-related parameters increases the performance of the staging system.

In conclusion, the present study supports the use of pre-treatment ALI, an easily measurable inflammatory/nutritional index, in daily clinical practice to improve prognostic stratification in surgically treated HPV-negative HNSCC.

## Data Availability

Yes.

## Appendix

**Table 5:** Definition of inflammatory and nutritional indexes

Index	Formula
ALI	$\mathrm{BMI}\cdotp \mathrm{Albumin}\cdotp \frac{\mathrm{Lymphocytes}}{\mathrm{Neutrophils}}$
PNI	10 · Albumin + 0.005 · Lymphocytes
SIM	$\frac{\mathrm{Neutrophils}\cdotp \mathrm{Monocytes}}{\mathrm{Lymphocytes}}$
